# Grip on challenging behavior: process evaluation of the implementation of a care program

**DOI:** 10.1186/1745-6215-15-302

**Published:** 2014-07-25

**Authors:** Sandra A Zwijsen, Martin Smalbrugge, Jan A Eefsting, Debby L Gerritsen, Cees MPM Hertogh, Anne Margriet Pot

**Affiliations:** Department of General Practice and Elderly Care Medicine/EMGO + Institute for Health and Care Research, VU Medical Center, Amsterdam, the Netherlands; Zonnehuisgroep IJssel-Vecht, Zwolle, the Netherlands; Department of Primary and Community Care: Centre for Family Medicine, Geriatric Care and Public Health, Radboud University Nijmegen Medical Centre, Nijmegen, the Netherlands; Department of Clinical Psychology, Faculty of Psychological and Educational Sciences, EMGO + Institute for Health and Care Research, VU University, Amsterdam, the Netherlands; Netherlands Institute of Mental Health and Addiction, Utrecht, the Netherlands

**Keywords:** Nursing home, Dementia, Behavior, Process evaluation, Implementation, Intervention

## Abstract

**Background:**

The Grip on Challenging Behavior care program for managing challenging behavior was implemented in the dementia special care units of 17 Dutch nursing homes. A process evaluation of the implementation of the care program was performed to determine the quality of the implementation and the lessons to be learned for future implementation.

**Methods:**

The care program was implemented according to a stepped wedge design. First-order data (data on recruitment, reach, relevance and feasibility) were used to determine the validity of the study, and second-order data (intervention quality and the barriers and facilitators for implementing the care program) were used to describe the implementation process. Two structured questionnaires were administered to care staff and key stakeholders and semi-structured interviews were held in the units.

**Results:**

University affiliated and non-affiliated nursing homes from different parts of the Netherlands participated. The resident participation rate was over 95% and the participation rate for the training sessions was 82%. Respondents considered the care program relevant and feasible. The degree of implementation was not optimal. The barriers and facilitators in implementing the care program could be divided into three categories: organizational aspects, culture on the unit and aspects of the care program itself.

**Conclusions:**

The recruitment, reach, relevance and feasibility are sufficient to allow for analysis and generalization of the effects of the care program, but the degree of implementation should be taken into account in further analysis. Future projects that involve implementation should consider the specific features of the organization and the cultural orientation of the unit to better adapt to specific needs.

**Trial registration:**

The Netherlands National Trial register under number NTR2141 registered on 11 December 2009. Randomization took place in November 2010, and the first intervention group started using the intervention in February 2011.

**Electronic supplementary material:**

The online version of this article (doi:10.1186/1745-6215-15-302) contains supplementary material, which is available to authorized users.

## Background

Challenging behavior, like aggression or wandering, is a major issue in nursing homes for people with dementia. Over 80% of residents of dementia special care units (DSCUs) show some form of challenging behavior [1], which affects both the quality of life of residents [2] and the (mental) health of nursing staff [3]. A structured way to detect, analyze, treat and evaluate treatment of challenging behavior is often lacking [4]. Therefore, in the Grip on Challenging Behavior study, a care program was developed that offers a stepwise and structured approach to the management of challenging behavior [5]. To determine the effects of the care program, it was implemented in several Dutch nursing home wards.

In the nursing home setting, much effort is being made on improvement of care. Next to the Grip on Challenging behavior project, projects to improve care for residents with depression, to improve medication administration, to prevent pressure ulcers and to better detect and treat pain are just a few other examples of recent attempts to establish evidence-based care of high quality [6–9]. Implementing such new interventions in nursing homes is difficult, for the structure and culture of the nursing home setting is complex and heterogeneous. Therefore, as several researchers have pointed out already, information about the degree of implementation in such studies is crucial for their credibility [6, 10, 11]. For example, in all of the intervention studies mentioned above, the implementation of the strategies to improve care was complicated and not always completely successful, which has implications for both the interpretation of the trial results and the implementation in actual practice; after all, if a study lacks internal validity (either due to insufficient sample size or poor implementation of the intervention), analysis on the effects will be meaningless [6]. Also, knowledge on sampling and the quality of the intervention is important for the applicability of the findings in clinical practice. For clinicians and policy makers, applicability in practice and knowledge on implementation barriers and facilitators are of critical importance in their decision making. In other words, to ascertain true contribution of the intervention to actual practice, a process evaluation of the implementation of the Grip on Challenging Behavior care program is needed.

Although there is no consensus about the ideal method, several attempts have been made to make a general framework for process evaluation of implementation of interventions [10, 12, 13]. Generally, these frameworks include ways to determine both internal validity (for example, recruitment of participants, reach of the intervention, actual use of the intervention) and external validity (for example, feasibility, acceptability). Recently, Leontjevas and colleagues [6], following earlier theories on process evaluation of implementation of interventions [11, 14, 15], proposed a model of first- and second-order process evaluation, which distinguishes between first-order process data that assess sampling and intervention quality (internal and external validity; relevant for analyzing effects and interpreting of results) and second-order process data that concern knowledge on the barriers and facilitators for the implementation of the intervention (relevant for future implementation) [6]. For this paper, this model of first- and second-order process evaluation will be used to report on the implementation of the Grip on Challenging Behavior care program. The aim of this paper is to determine the internal and external validity of the research conducted and to gain knowledge on barriers and facilitators for future implementation.

## Methods

The process evaluation for Grip on Challenging Behavior was conducted during the implementation and the research into the effects of the care program. The methods of the effect study are described in detail elsewhere [5].

### Design

**Figure 1:**
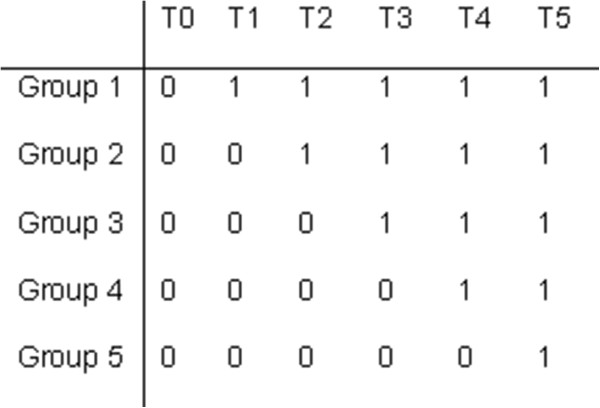
**Stepped wedge design.** 0, control condition (usual care); 1, intervention (care program); T0-T5, measurements, each four months apart. Each group consists of three or four dementia special care units.

The care program was implemented according to a stepped wedge design. By using this design, the participating DSCUs were randomly assigned to five intervention groups, which received the intervention at different time points. Measurements on challenging behavior, quality of life, psychoactive drugs and restraints took place every 4 months as part of the effect study; after each measurement, a new group of DSCUs was trained and started to use the care program (Figure 1). This resulted in a measurement period of 20 months (February 2011 to October 2012); after 16 months all DSCUs used the care program.

### Ethics

The study protocol [5] is in accordance with the declaration of Helsinki and with the Dutch legislation on medical research and it is in agreement with the Conduct Health Research of the Dutch federation of Biomedical Scientific Societies. The Grip on Challenging Behavior study was approved by the Medical Ethics Review Committee of the VU Medical Center. In accordance with Dutch legislation, the study can be performed without a review procedure by the committee and without obtaining informed consent of the (representatives of) the resident, because in the study only observational data gathered by nursing staff as part of their daily work were used. However, all legal representatives of the residents were informed about the study and were given the opportunity to object to data of their proxy being used for research purposes at any time during the study.

The participants for the interviews on the implementation of the care program gave their informed consent for being audiotaped and for their statements being used in the evaluation of the implementation.

### Participants

Although Dutch nursing homes typically house older people with mental or physical disabilities, they differ in, for example, the way care is organized, staff-patient ratio and employment of various disciplines [16, 17]. In the Netherlands, nursing home care is divided into care for people with predominantly somatic illnesses (somatic units) and care for people with dementia or dementia-like disorders (DSCUs). Most nursing homes contain both somatic units and DSCUs. For this research project, only DSCUs of regular nursing homes were approached for participation.

In these DSCUs, care is provided by nursing staff with different levels of training in care-giving. Nursing assistants (who have had 2 years of training on care-giving and supporting people with personal care and housekeeping) are involved in daily care tasks, such as helping residents in and out of bed and assisting them with toileting. Certified nurse assistants (who have received 3 years of training on care-giving and nursing skills) are also involved in medical care, such as wound care and administering medication. Certified nurse assistants can also be certified to function as a responsible contact person for the resident, the responsible contact person is involved in the development and implementation of the individual care plan. A team leader (a registered nurse or a certified nurse assistant who also have had management training) is responsible for the day-to-day functioning of the care team. A psychologist and an 'elderly care physician' [18], who have a permanent position in Dutch nursing homes, are also part of the care team. Also, in some nursing homes, a registered nurse is also part of the care team. For the Grip on Challenging Behavior care program, every member of the care team (psychologist, physician, team leader, nursing staff) were invited to the training sessions, regardless of education level (trainees and temporary staff were also invited).

For each participating DSCU, a contact person was appointed whom the researchers could contact for updates on the implementation process and who could be contacted to make appointments for interviews. In most cases, the team leader was the contact person, but the psychologist of the DSCU or one of the care staff members with an executive function could also function as contact person.

### The care program

**Figure 2:**
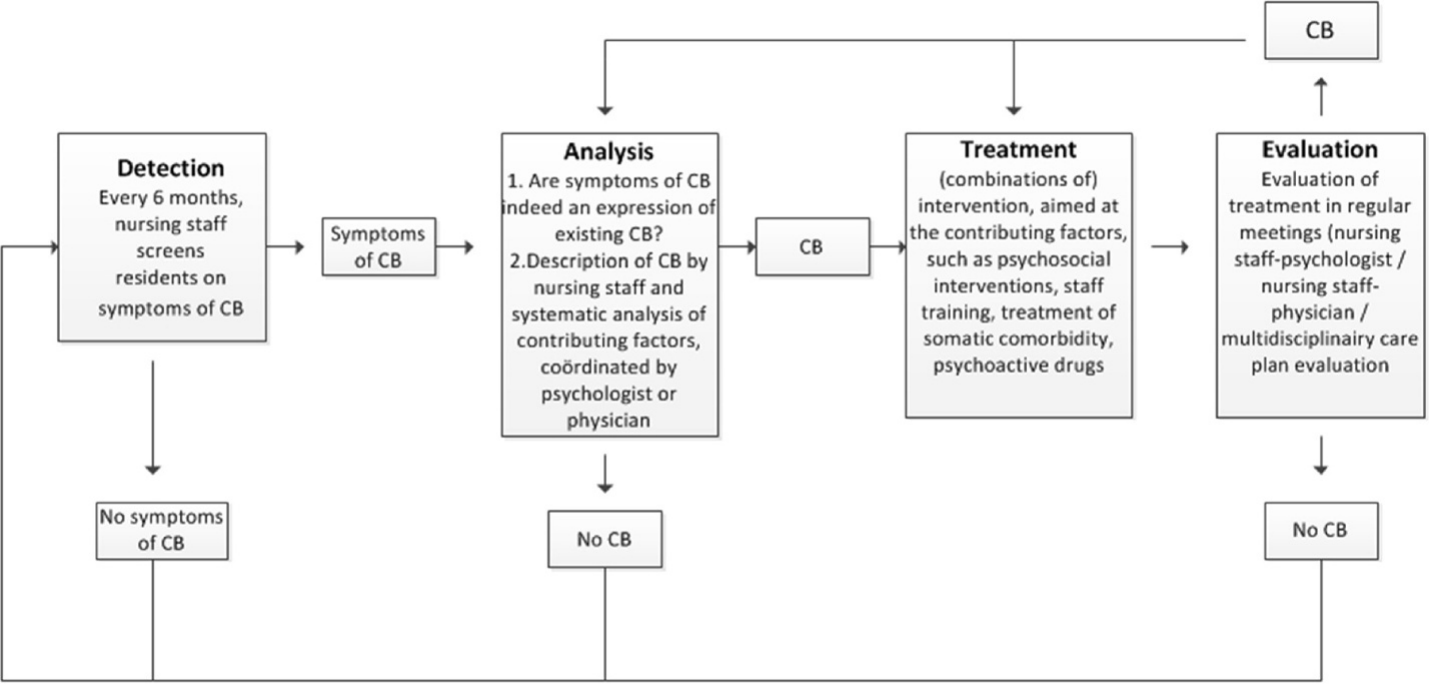
**Outline of the care program.** CB, challenging behavior.

The Grip on Challenging Behavior care program consists of four steps (Figure 2). The full content of the care program is described elsewhere [19].

The first step is 'detection'. Challenging behavior is usually detected by the care staff and reported to either the psychologist or the elderly care physician. In the care program, this is called ‘spontaneous observation’. To prevent challenging behavior being overlooked, the use of a screening tool is also outlined in the care program. Every 6 months, prior to the multidisciplinary care meeting, the care staff fills in the Neuropsychiatric Symptoms Inventory questionnaire [20] to detect challenging behavior.

The second step is 'analysis'. When care staff detect challenging behavior (either spontaneous or via the screening tool), the care program assists care staff to conduct a structured analysis by a form containing various questions concerning the challenging behavior (for example, what does the behavior look like, to whom is it challenging, where does it take place, and so forth). It was emphasized in the training that every care staff member could spontaneously detect signs of challenging behavior. Once the analysis starts, the certified nurse assistant who is the responsible contact person for that resident becomes involved and agreements are made about who should be involved in the follow-up process. Next, the care staff calls in and hands over their completed form to either the elderly care physician when they suspect a physical cause of the behavior, or to the psychologist in case a psychosocial cause is more likely. Within the care program, both the physician and the psychologist have their own analysis form, based on the guidelines of their own discipline. The physician and psychologist can consult or refer to one another if necessary. The analysis ends with a thorough description of the behavior and the probable causes.

The third step is the 'treatment'. Treatment should be focused on the (probable) causes identified during the analysis and can be made up of various components, such as education, psychosocial support, treatment of physical causes, psychosocial interventions, and so forth. The treatment plan consists of a treatment goal, the interventions to obtain this goal and the planning of an evaluation. The treatment plan is outlined on the treatment form and the current situation is rated on a ten-point visual analogue scale. The rating scale is not an objective tool but it can be used to quantify feelings of severity of both the behavior or the disruption it causes (that is, resident does not pace at all = 1; resident is constantly pacing = 10). At the bottom of the form, the evaluation date is planned.

The fourth and final step is the 'evaluation'. The care program provides a flow chart that should be passed through during evaluation. Again, the current situation is rated on a scale from 1 to 10 to see if there is any improvement.

At the start of the implementation, all care staff, including the psychologist and physician, receive a total amount of 1 day training (split into two sessions). In the training, causes and mechanisms of challenging behavior are discussed and the use of the care program is explained.

#### Process evaluation

**Figure 3:**
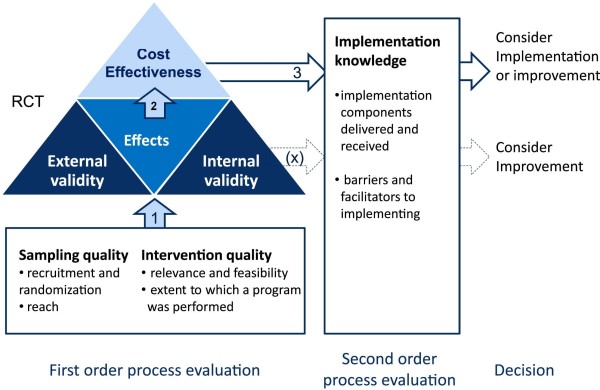
**Framework of the first- and second-order process evaluation.** Sampling quality: description of recruitment and randomization procedures and attendance rate of the training sessions. Intervention quality: indicators for feasibility and relevance of the care program and measurement of the use of the separate steps in the care program. Implementation knowledge: description of the number of implementation components provided and received and a description of the factors that (could) have influenced implementation. If first-order process data confirm the validity of the study (1), effect and cost-effectiveness analysis may be used (2) together with implementation knowledge (3), for further implementation or improvement of the care program. If validity is limited, implementation knowledge might be used to improve the care program (X). Adapted from Leontjevas and colleagues [6] RCT, randomized controlled trial.

As described earlier, the model of first- and second-order process data described by Leontjevas and colleagues [6] was used to conduct the process evaluation (Figure 3). First-order process data consider the sampling quality (recruitment, randomization and reach; external validity) and the intervention quality (relevance and feasibility of the care program and the extent to which the program was implemented; internal validity), and second-order data consider information on implementation (implementation components delivered and received and barriers and facilitators). Ideally, first-order data should be evaluated before analysis of the actual effects of an intervention since the outcome of this evaluation can be used to correct or complete the analysis. Second-order process data are more important for future implementation research and future implementation of the care program. Although it is possible to evaluate second-order process data at a later stage, in this paper both first- and second-order process data are presented together to get a complete picture of the implementation and the quality of the trial.

#### First-order process data

The sampling quality was determined by a description of the recruitment of the DSCUs, the DSCU randomization procedure and the reach (proportion of care staff receiving the training). The intervention quality (relevance, feasibility and extent to which the program was performed) was determined with two separate questionnaires. After the second and third measurement in the effect study, the certified nurse assistants who were first contact persons for particular residents that were in the first and second intervention group (seven DSCUs) received a short questionnaire (Q1) (n = 56) about their expectations and appreciation of the care program (relevance and feasibility). The questions used for this evaluation were: ‘what do you think of the structure of the care program (bad, not good, good, very good)?’ and ‘how much faith do you have in the care program being able to decrease challenging behavior on your DSCU (rating 1 to 10, 1 no faith at all; 10 being convinced the care program will be able to decrease challenging behavior)’. Next to this, a more extended digital questionnaire (Q2) (Additional file 1) was distributed among all team leaders, psychologists and elderly care physicians at the end of the study (n = 48, representing 16 DSCUs - the 17th DSCU moved to another location during the study and was therefore not included in any further analyses). This questionnaire contained items with pre-arranged answer categories (for example, in which percentage of all cases with challenging behavior is this form used: <25%; 25-50%, 50-75%, >75%?) and items with an open response (for example, what were the barriers for implementation?). People either completed the questionnaire themselves or it was completed by one of the researchers who held a telephone interview with those participants who had not yet responded to the written invitation to fill in the questionnaire. Q1 and Q2 were both based on earlier research of Leontjevas and colleagues [6]. Descriptive functions of SPSS 20.0 [21] were used for analysis of the data.

#### Second-order process data

Two methods were used describe the second-order process data of implementing the care program. The extended questionnaire described above (Q2) also contained open questions about the barriers and facilitators in implementing the care program. Next to this, one of the researchers (SAZ) held interviews for evaluation purposes with staff of the participating DSCUs. For the interviews, a topic list was used which contained topics on the feasibility and implementation of the several different steps of the care program and on the implementation process of the care program as a whole. The interviews took between 10 and 45 minutes (depending on the involvement of the participant in the implementation process). In total, 51 interviews were held with 29 members of nursing staff (nursing assistants and certified nurse assistants), one recreational therapist, 12 physicians, 15 psychologists and seven team leaders (some interviews were held with more than one person). All these interviews were audiotaped and transcribed verbatim. Two of the researchers (SAZ and MS) analyzed the open questions in Q2 on re-occurring themes with regards to the barriers and facilitators in implementing the care program. Subsequently, directed content analysis was used to confirm the themes that were found in Q2 in the transcripts of the interviews [22].

## Results

### First-order process data

#### Sampling quality

**Recruitment and randomization** The University Network of Organisations for Care for the Elderly of the VU University Medical Center and the University nursing home network of the Radboud University Nijmegen Medical Center invited the affiliated nursing homes to let one of their DSCUs participate in the research project. These networks consist of 32 care organizations. Seven were not invited because they were already involved in other research projects. Thirteen organizations did not respond, whereas 12 did. To gain enough participants, convenient sampling was used to recruit eight further nursing homes.

**Figure 4:**
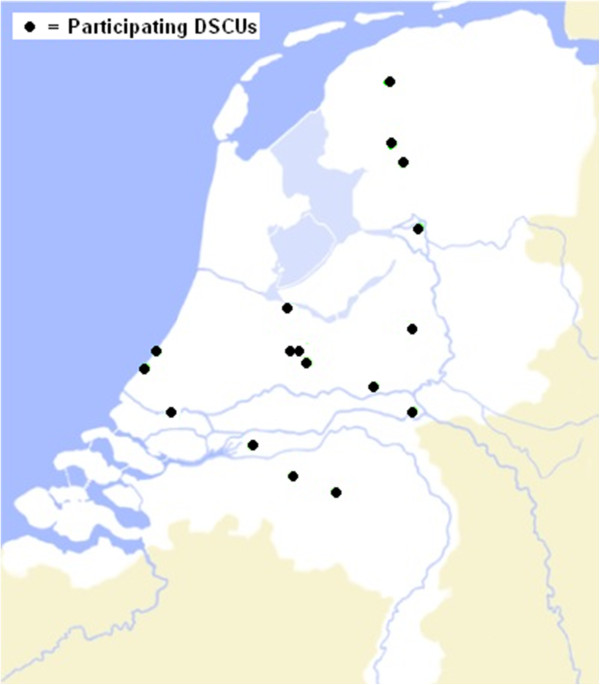
**Map of the distribution of participating dementia special care units (DSCUs) in the Netherlands.**

Of the twenty organizations that responded four organizations eventually decided not to take part, because of organizational changes in the nearby future (three), or because of involvement in another new approach for management of behavioral problems (one). The participating organizations were free in selecting one of their DSCUs for participation, although DSCUs for special groups (for example, Korsakov, young-onset dementia) were excluded. On organization chose to select two participating DSCUs that were part of different locations. Of the 17 participating DSCUs, nine were located in the densely populated Randstad area of the Netherlands, and the other eight were situated in less densely populated areas (Noord-Brabant, Gelderland and Friesland) (Figure 4). All DSCUs were split up into several shared living rooms in which a group of residents had their regular place. The mean size of the DSCU was 29 residents (minimum 18, maximum 43) and a mean number of 11 (minimum 6, maximum 19) residents resided in one living room. One DSCU dropped out after T4 because it moved to another location. All other units participated in the study from the first measurement (T0) until the last (T5). Randomization took place using random allocation software [23] in November 2010. To avoid contamination, block randomization was used for two DSCUs which were part of one larger organization (the two units were entered as one in the software). All other DSCUs stemmed from separate organizations.

**Reach** Legal representatives of the residents were informed about the research project and the possibility to object to the use of observational data through a folder and a letter from the DSCU leader. This resulted in a participation rate of a minimum 89% (42/47) and maximum 100%. All residents were included in the implementation of the care program as the care program was implemented at the unit level rather than the resident level.

Before the start of the intervention, the contact person of the DSCU provided a list of all care staff working at the DSCU. All staff were invited to the training, which was made compulsory by the DSCU leader. Care staff received a certificate when they had participated in both the trainings session, or when they had made up for their absence during one of the trainings sessions by reading the education material and gaining information through co-workers. In other words, when the researchers were convinced a staff member had obtained enough training to understand the background of the care program and to be able to use the care program in care practice, a certificate was granted. The participation rate was calculated by comparing the number of invitations for the trainings sessions to the number of certificates granted; this resulted in a mean rate of care staff of 81% (SD 14; range 34 to 97%). With regards to the psychologists and physicians, all but three psychologists attended both trainings sessions, and three psychologists attended one of the two sessions. All but five physicians attended both trainings sessions, four physicians attended one of two trainings sessions and one physician received information about the care program in an individual session.

The main reasons for not participating were being on leave and illness.

#### Intervention quality

**Relevance** Q1 had a response of 60% (9/15) on T1 and 56% (23/41) on T2. In response to the question ‘What do you think of the structure of care program?' one responder (3%) answered ‘not good’, 26 (81%) responded with ‘good’ and five responders (16%) answered ‘very good’. The overall score for the confidence in the program being able to diminish challenging behavior on the DSCU was 6.6 (SD 0.9).

The response to Q2 was 85% (41/48 questionnaires, 35% telephone interview, 65% digital questionnaire, of which one person did not fill in the open questions about barriers and facilitators). The 15% that did not respond were either no longer working on the DSCU or they had only recently started working on the DSCU. In each participating DSCU at least two out of three ‘key figures’ (physician/psychologist/team leader) responded. On the question on satisfaction with the content of the care program (0 = not satisfied, 1 = hardly satisfied, 2 = slightly satisfied, 3 = satisfied, 4 = very satisfied), the most common answer was ‘satisfied’ (for example, median = 2).

**Feasibility** Most responders to Q2 (27/41) stated that the care program could be used in the currently available time. Some of the advantages about working with the care program that were mentioned were ‘the process is more clear, there is more structure’, ‘better analysis of behavior’ and ‘earlier detection, more attention for behavior’. The most frequently mentioned disadvantage (23/41) was ‘too many forms’. Some responders (12) also answered ‘big time investment’. All but one responder would recommend the use of the program to colleagues, although three responders stated that they would only advise on the use of some parts of the program.

**Extent to which the program was performed** In Q2, responders were asked whether the forms of each step of the care program were being used, and whether they used the care program in all cases of challenging behavior. This question (which percentage of all cases of challenging behavior is treated according to the care program?) was asked with regard to the analysis forms in step 2, the treatment form in step 3 and the evaluation form in step 4; the analysis form for care staff was best implemented, and the treatment and evaluation forms were the least used form (Table 1).

**Table 1:** **Use of the forms of the care program in case of challenging behavior per dementia special care unit**

	Never	<25%	25-50%	51-75%	>75%
Analysis care staff form	0	0	3	8	5
Analysis physician/psychologist form	0	2	2	10	2
Treatment form	2	6	4	4	0
Evaluation form	1	8	5	2	0

Data from 16 dementia special care units and 41 respondents; % of the residents with challenging behavior (pre-arranged answer categories, answers derived from more than one key person on the dementia special care unit).

### Second-order process data

#### Implementation components

At the start of the intervention, two training sessions were held. In the first session, information was given about causes and mechanisms of challenging behavior and the use of the care program was explained. The second session was held approximately 2 weeks after the first session. During these 2 weeks care staff had practiced using the care program. In the second session, feedback about the use of the care program was discussed.

To further facilitate the implementation, one of the researchers (SAZ) arranged evaluation sessions with the involved care staff, DSCU leader, psychologist or physician. In these evaluation sessions, barriers and facilitators in implementing the care program were discussed and tailored communication was used to improve implementation in the DSCU [24]. Every DSCU was visited at least once for an evaluation session; the follow-up of this session could be either by telephone, email, or with another evaluation session, depending on the degree to which the implementation was already successful. In total, 45 evaluation sessions with tailored communication were held (range 1 to 5 per DSCU).

Also, one of the researchers (SAZ) could be contacted via telephone or email if there were any questions with regards to the care program. This option was rarely used; two psychologists and one team leader took the initiative in asking a question regarding the content of the care program via email. Telephone and email were mostly used for requests to send more forms.

#### Barriers and facilitators for implementation

From the answers on open questions in Q2, several categories of barriers and facilitators emerged which were confirmed in the analysis of the transcripts of the interviews. The categories can be divided into three themes: organizational aspects, culture of the DSCU and the layout of the care program.

##### Organizational aspects

*Staff turnover* It became apparent from the interviews that staff turnover rates could influence the implementation process. Staff turnover sometimes resulted in situations in which only a part of the team was truly well informed about the care program. Although attempts were made to train new staff members, the situation remained suboptimal. While the turnover of nursing staff had adverse consequences, the change of DSCU leader, psychologist or physician was even more detrimental, for they had a leading role in implementing the care program. When these key stakeholders were absent for a period, there was often a drop in attention for implementing the care program. When key stakeholders were then replaced, the new person would often need time to get acquainted with the use of the care program as well as all other methods used on the DSCU, which greatly slowed down the implementation. Overall, there were no DSCUs without change in key stakeholders; there was a mean turnover of 2.64 key persons (range 1 to 6) per DSCU. Absence or change of these key persons was a real barrier in implementing the care program, as this psychologist points out in one of the interviews:

*Psychologist:*

*Well, for example, I drew up a plan for this lady. And in my absence, a physician, a new physician, just crossed right through it.*

*High workload* High workload and time being scarce were often mentioned as one of the barriers to implementing the care program. Although opinions differed on the amount of time the care program would really cost once implemented, it was obvious that having to learn to work according to the care program would cost some time, which was, in the eyes of care personnel, not always available.

*Nurse:*

*But we work under a constant lack of time and staff shortage. And these kinds of things are the first to slip through then.*

*Psychologist:*

*Yes, well, really the time pressure, yeah that’s it. And then also my own involvement… I realize I’m not at the unit very often and I kind of feel like, please don’t use it [the care program], because I can’t handle anything extra at the moment. And well, I think that is alarming, because that is a very ambiguous signal that you are sending*

*Concurrent and former projects* It appeared that implementation of the care program was easier on DSCUs that rarely initiated new projects. Key persons in these DSCUs stated that being cautious not to adopt too many new projects helped in keeping care staff motivated when a new project was proposed. In contrast, some DSCUs were involved in several new projects, such as implementing electronic health records for all residents or using new forms for quality improvement on the DSCU. This seemed to interfere with implementing the care program, as time was already scarce. Also, some of the staff members of those DSCUs expressed skepticism about new projects. They had seen many new projects come and go during the last years, many of which did not cause relevant improvement to daily care.

*Psychologist:*

*Yeah, well, it makes a difference that we are not, well, this is a fairly new location, where they have not started up all kind of new projects, which does make a difference you know.*

*Multidisciplinary meetings* For the care program to work properly there has to be a structure in which physician, psychologist and care staff meet each other regularly. Although this happens during the (obligatory) multidisciplinary care meetings, it was a precondition for implementing the care program that there would be extra time in which the forms of the care program would be discussed. In reality, this precondition was not always met. The working hours of the physician or psychologist did not always correspond with each other or with the care staff that completed the form. Because of the lack of contact between disciplines, a resident was sometimes treated by more than one discipline without interdisciplinary consultation and with one of the disciplines not using the care program. Also when care staff completed an analysis form it sometimes took many weeks before a psychologist or physician was able to respond to it; this did not encourage care staff to complete more forms in the future.

*Organizational changes* During the implementation of the care program, some of the DSCUs encountered minor or major organizational changes. For example, in one of the DSCUs there were plans at hand that would change the position of several staff members. Another organization changed their management structure, which caused changes in responsibilities and duties of DSCU leaders. Such changes cause turmoil on DSCUs which interferes with the implementation of the care program.

##### Culture of the organization/DSCU

*Support of key persons* For a rapid and solid implementation process it was important that key persons such as physicians, psychologists and DSCU leaders functioned as ‘team champions’ in supporting the use of the care program [25]. These team champions could support the implementation by embracing the care program and emphasizing to care staff that they complete the forms when they report challenging behavior, and by reporting back on the forms or helping complete them when care staff found it difficult. Without one or more key persons taking the lead on implementation and on stimulating the care staff to use the forms, it was very difficult to keep everyone focused on using the care program. Also, support of higher management of the organization (for example, by calculating in extra time) facilitated the implementation, because more time and understanding were available during implementation.

*Attitude towards change* In the individual interviews, some respondents stated that their team was very open to a new method in managing behavioral problems. These teams often seemed to be motivated to start working with the Grip on Challenging Behavior care program. In other DSCUs, respondents observed there was more reluctance in changing current routines and procedures. This was also noticed by DSCU managers and sometimes by psychologists and physicians.

*Certified nurse assistant:*

*People are often stuck in the old system. They do not always want to try out new things. But if you save time later on, that affects the resident I think.*

##### Aspects of the care program

*The care program was not digitally available* Some of the organizations of which the DSCUs were part had recently transferred to using electronic health records. Part of this transfer was to eliminate all paper files and forms, so as to create one method of working. Because the digital systems are different for almost every nursing home, it was not possible to provide one general digital version of the care program and it was therefore only provided as a paper version. For those DSCUs that only had a digital administration systems, the paper forms of the care program became easily forgotten. Also, the work method of using forms did not fit in with the normal working methods, which was a barrier for the implementation.

*Many forms* The care program consists of eight different forms (detection tool, three analysis forms (nursing staff, psychologist, physician), an extra analysis tool for the psychologist, a treatment form, an evaluation form and one agenda form to overview the process). Although the use of the forms was separated by different disciplines and time periods, many respondents complained that, at first sight, the number of forms was overwhelming and that this made it tempting to discuss behavior informally or via email instead of starting to fill in an analysis form. When asked, however, respondents often stated that almost all forms were useful and completing the forms did not take much extra time after all. Even so, merely the first impression and the prospect of having to complete the forms did hinder the implementation.

*Team leader:*

*The only thing that does not really work as an advantage, although you do really need all, is the number of forms. And I think that when you just put it out there, like ‘these are the forms’… that that can scare people off.*

## Discussion

The aim of this paper was to describe the process of implementing the Grip on Challenging Behavior care program in 17 DSCUs. The model of Leontjevas and colleagues [6] was used to evaluate both first-order and second-order process data.

### First-order process evaluation

Data on the sampling quality show that the participation rate of residents and the rate of staff receiving training sessions was over 80%. The 17 participating nursing homes were not randomly selected, but the variance in size and location allow for generalization of the study effects [26].

The respondents considered the structure of the care program to be good and they generally believed the care program could diminish challenging behavior in their DSCU. The actual degree of implementation was not optimal; in only a small percentage of the DSCUs were all forms of the care program used, and none of the DSCUs used all forms in all cases of challenging behavior. Obviously, the later steps in the process are the first to be omitted, as earlier research on a stepwise approach to depression also confirmed [6]. In contrast, all DSCUs used at least the first two steps of the care program (detection and analysis), which probably still resulted in adjustment in the individual care plan although the treatment form was not used.

The degree of implementation should be considered in the analysis. If possible, analysis should be corrected for degree of implementation, or subgroup analysis should be performed to analyze differences in effects for different degrees of implementation.

### Second-order process evaluation

The Grip on Challenging Behavior care program was developed as a practical tool that meets the needs of those working with challenging behavior in nursing home care. Although education theory emphasizes that professionals are more prone to adapt innovation when it is based on problems they encounter in actual practice [27], the implementation of the care program was not optimal. Despite the use of several implementation strategies (training, tailored communication, telephone and email support), analysis of the second-order process data identified various barriers in implementing the care program.

Organizational aspects influenced the ease with which the care program was embraced in a DSCU. Staff turnover, high workload, concurrent projects, cancelled meetings and organizational changes were described as barriers for implementing the care program. It is not the first time these organizational factors have been found to be of influence on the implementation of an intervention in nursing home care [7]. It seems that, although the extent of the project and the time investment is explained before the start of the project, the decision to participate in a project is often made by managers, without consultation of team members of the DSCU. This top-down decision making process might lead to an imbalance between the admittance of the care program in the policy of an organization and the possibilities of actual implementation in a specific DSCU. For example, most DSCU leaders know the turnover rates and the amount of care staff working on temporary or flexible contracts in their DSCU. The absence of a permanent care staff team makes it almost impossible to implement any changes in the nursing home setting. It thus seems of great importance to consult not only the management team of an organization, but also the DSCU team that is involved in the implementation of the care program in the decision-making process. Although it is impossible to be ahead of all future organizational changes, the DSCU team can assess the possibility that organizational aspects such as staff turnover and concurrent projects will form barriers in implementation.

Organizational aspects thus sometimes appeared to be a barrier in implementing the care program. The culture in the DSCU and organizational aspects such as staff turnover, organizational changes and involvement in concurrent projects strongly interact. Not surprisingly, the interviews showed that DSCU culture could also form a barrier as well as a facilitator in the implementation process. The way in which care staff dealt with the introduction of the care program can be explained through the four cultural orientations that can be distinguished from the competing values framework [28, 29]. The first, group culture, is characterized by strong social relations and an internal focus. This type of culture might be linked to the reluctance to change found in some DSCUs, since DSCUs with a group culture are focused on the internal organization of the team rather than on improving and changing working methods by adapting an external method [29]. The DSCU teams that were enthusiastic to start working with the care program seem to have a more open attitude towards change and welcomed external input, which is characteristic for the second cultural orientation, developmental culture*.* Rational culture is control oriented and focuses on productivity and achievement. There were no DSCUs characterized by this orientation, which might be only logical in a non-profit organization. Finally, hierarchical culture emphasizes stability and is characterized by uniformity, internal efficacy and a close adherence to rules and regulations. For DSCUs with this orientation, the attitude of the key persons in implementing the care program is crucial. Earlier research on implementation of a multifaceted intervention in nursing homes also showed that having a team champion (for example, someone who is passionate about the use of the care program) has a substantial impact on the effectiveness of a team to adapt to innovation [9]. Implementation indeed seemed to be facilitated when an enthusiastic key person was willing to commit to the care program and that absence or departure of such a team champion seriously impacted the implementation process.

Finally, two aspects of the care program formed a barrier in the implementing the care program. The number of forms to be filled in scared some people off. Also, a digital version of the care program would have been more appropriate for some DSCUs. For future implementation of the care program, a reduced number of forms (that is, merging some forms together) and digitalizing of the forms should be considered.

## Conclusion

The first-order process data allow analysis of the effects of the care program, although the degree of implementation should be considered. With regard to the second-order data, the barriers in implementing the care program can partly be overcome by reshaping some components of the care program, but the major implementation issues involve the organizational culture of the DSCUs. Future projects that involve implementation should involve leaders of care teams in the decision to participate. It would also be well advised to perform a diagnostic analysis [27] of organizational aspects and organizational culture before the start of the project, to better adapt to the specific needs and possibilities within an organization.

## Electronic supplementary material

Additional file 1: **Questionnaire for evaluation of the implementation of the care program.** (PDF 59 KB)

## Abbreviations

DSCU: dementia special care unit
Q1: questionnaire 1 (questionnaire containing three questions about the expectations of the care program)
Q2: questionnaire 2 (questionnaire for evaluation of the implementation of the care program).

## References

[1] Zuidema S, Koopmans R, Verhey F (2007). Prevalence and predictors of neuropsychiatric symptoms in cognitively impaired nursing home patients. J Geriatr Psychiatry Neurol.

[2] Van de Ven- Vakhteeva J, Bor H, Wetzels RB, Koopmans RT, Zuidema SU (2013). The impact of antipsychotics and neuropsychiatric symptoms on the quality of life of people with dementia living in nursing homes. Int J Geriatr Psychiatry.

[3] Schmidt SG, Dichter MN, Palm R, Hasselhorn HM (2012). Distress experienced by nurses in response to the challenging behaviour of residents - evidence from German nursing homes. J Clin Nurs.

[4] Dorland LM, Pot AM, Verbeek MA, Depla M (2007). [Psychological assistance for older people in care homes and nursing homes; substudy 7] [in Dutch].

[5] Zwijsen SA, Smalbrugge M, Zuidema SU, Koopmans RT, Bosmans JE, van Tulder MW, Eefsting JA, Gerritsen DL, Pot AM (2011). Grip on challenging behaviour: a multidisciplinary care programme for managing behavioural problems in nursing home residents with dementia. Study protocol. BMC Health Serv Res.

[6] Leontjevas R, Gerritsen DL, Koopmans RT, Smalbrugge M, Vernooij-Dassen MJ (2012). Process evaluation to explore internal and external validity of the "Act in Case of Depression" care program in nursing homes. J Am Med Dir Assoc.

[7] Stuijt CC, Klopotowska JE, Kluft-van DC, Le N, Binnekade J, van der Kleij B, van der Schors T, van den Bemt P, Lie-A-Huen L (2013). Improving medication administration in nursing home residents with swallowing difficulties: sustainability of the effect of a multifaceted medication safety programme. Pharmacoepidemiol Drug Saf.

[8] Plooij B (2012). (Under)treatment of pain in dementia [PhD Thesis].

[9] Sharkey S, Hudak S, Horn SD, Barrett R, Spector W, Limcangco R (2013). Exploratory study of nursing home factors associated with successful implementation of clinical decision support tools for pressure ulcer prevention. Adv Skin Wound Care.

[10] Saunders RP, Evans MH, Joshi P (2005). Developing a process-evaluation plan for assessing health promotion program implementation: a how-to guide. Health Promot Pract.

[11] Steckler A, Linnan L (2002). Process Evaluation for Public Health Interventions and Research.

[12] Glasgow RE, Vogt TM, Boles SM (1999). Evaluating the public health impact of health promotion interventions: the RE-AIM framework. Am J Public Health.

[13] Baranowski T, Stables G (2000). Process evaluations of the 5-a-day projects. Health Educ Behav.

[14] Eldridge S, Ashby D, Bennett C, Wakelin M, Feder G (2008). Internal and external validity of cluster randomised trials: systematic review of recent trials. BMJ.

[15] Rothwell PM (2005). External validity of randomised controlled trials: "to whom do the results of this trial apply?". Lancet.

[16] Willemse BM, de Jonge J, Smit D, Depla MF, Pot AM (2012). The moderating role of decision authority and coworker and supervisor support on the impact of job demands in nursing homes: a cross-sectional study. Int J Nurs Stud.

[17] Willemse BM, Smit D, de Lange J, Pot AM (2011). Nursing home care for people with dementia and residents' quality of life, quality of care and staff well-being: design of the Living Arrangements for people with Dementia (LAD)-study. BMC Geriatr.

[18] Koopmans RT, Lavrijsen JC, Hoek JF, Went PB, Schols JM (2010). Dutch elderly care physician: a new generation of nursing home physician specialists. J Am Geriatr Soc.

[19] Zwijsen SA, Gerritsen DL, Eefsting JA, Hertogh CM, Pot AM, Smalbrugge M (2014). The development of the Grip on Challenging Behaviour dementia care programme. Int J Palliat Nurs.

[20] Kaufer DI, Cummings JL, Ketchel P, Smith V, MacMillan A, Shelley T, Lopez OL, DeKosky ST (2000). Validation of the NPI-Q, a brief clinical form of the Neuropsychiatric Inventory. J Neuropsychiatry Clin Neurosci.

[21] IBM Corp. Released 2011 (2011). IBM SPSS Statistics for Windows, Version 20.0.

[22] Hsieh HF, Shannon SE (2005). Three approaches to qualitative content analysis. Qual Health Res.

[23] Sagheei M (2006). Random Allocation Software.

[24] Hawkins RP, Kreuter M, Resnicow K, Fishbein M, Dijkstra A (2008). Understanding tailoring in communicating about health. Health Educ Res.

[25] Shortell SM, Marsteller JA, Lin M, Pearson ML, Wu SY, Mendel P, Cretin S, Rosen M (2004). The role of perceived team effectiveness in improving chronic illness care. Med Care.

[26] Pot AM, De Lange J (2010). [Dementia housing monitor. A study into nursing home care for people with dementia.] [in Dutch].

[27] Grol R, Wensing M, Boscg M, Hulscher M, Eccles M, Grol R, Wensing M (2011). Theories on implementation. [Implementation. Effective improvements in patient care.] [in Dutch].

[28] Quinn R, Rohrbaugh I (1981). A competing values approach to organizational effectiveness. Publ Prod Rev.

[29] Stock GN, McDermott CM (2001). Organizational and strategic predictors of manufacturing technology implementation success: an exploratory study. Technovation.

