# Supplementary material for: Grip on challenging behavior: process evaluation of the implementation of a care program
Source: Trials. 2014 Jul 25;15:302. doi: 10.1186/1745-6215-15-302 (PMC4132912; doi:10.1186/1745-6215-15-302)
Supplement: Supplementary file 1 — Additional file 1: Questionnaire for evaluation of the implementation of the care program. (PDF 59 KB) [file 13063_2013_2183_MOESM1_ESM.pdf]

Evaluation Questionnaire Grip on Challenging Behavior

Because we have reached the end of the measurement period of the Grip on Challenging Behavior project we would like to ask you a couple of questions.

What is the name of your organization?

What is your function in this organization?

- Physician
- Psychologist
- Unit leader
- Different: _________

Question 1

Were you involved in during the full length of the full length of the project (01-feb-2011 till 01-nov-2012)?

- Yes
- No

If you answered “no”, when did you start working on the unit?

____

Question 2

To what degree, in your opinion, is the care program implemented on the unit?

- The care program is not or hardly being used on the unit (bad implementation)
- Some parts of the care program are being used (mediocre implementation)
- Most forms of the care program are being used (good implementation)
- All forms and steps of the care program are being used (excellent implementation)

Question 3

After the initial training sessions, how long did it take until the care program was being used to its full extent?

- I have not been employed long enough to answer this question (see question 1)
- The care program was used directly after the training sessions
- After approximately a month, we started working with the care program

- o After approximately 3 months, we started working with the care program
- o After approximately 6 months, we started working with the care program
- o After approximately 12 months, we started working with the care program
- o We did not start working with the care program ate any time

Question 4

Which forms of the care program a currently being used on the unit?

□ Agenda form

□ Detection tool

□ Analysis form care staff

□ Analysis form psychologist

□ Analysis form physician

□ Treatment form

□ Evaluation form

□ None of these forms

□ Different: ____________________

Question 5

In principle, the analysis form for care staff should always be filled in cases of challenging behavior. Could you indicate how often this is indeed happening in practice?

□ In 100% of the cases involving challenging behavior, the analysis form for care staff is being used

□ In 50-75% of the cases involving challenging behavior, the analysis form for care staff is being used

□ In 25-50 % of the cases involving challenging behavior, the analysis form for care staff is being used

□ In less than 25 % of the cases involving challenging behavior, the analysis form for care staff is being used

□ This form is never used

Question 6

After the detection of challenging behavior by care staff, the clinician should, in principle, fill in an analysis form. Could you indicate how often this is indeed happening in practice?

□ In 100% of the cases involving challenging behavior, the analysis form for the clinician (physician and/or psychologist) is being used

□ In 50-75% of the cases involving challenging behavior, the analysis form for the clinician (physician and/or psychologist) is being used

□ In 25-50 % of the cases involving challenging behavior, the analysis form for the clinician (physician and/or psychologist) is being used

□ In less than 25 % of the cases involving challenging behavior, the analysis form for the clinician (physician and/or psychologist) is being used

□ This form is never used

Question 7

When the analysis of behavior is completed, treatment can commence. According to the care program, the treatment form should be filled in. Could you indicate how often this is indeed happening in practice?

□ In 100% of the cases involving challenging behavior, the treatment form is being used

□ In 50-75% of the cases involving challenging behavior, the treatment form is being used

□ In 25-50 % of the cases involving challenging behavior, the treatment form is being used

□ In less than 25 % of the cases involving challenging behavior, the treatment form is being used

□ This form is never used

Question 8

To be able to eventually evaluate the treatment, the evaluation form can be used. Could you indicate how often this is indeed happening in practice?

□ In 100% of the cases involving challenging behavior, the evaluation form is being used

□ In 50-75% of the cases involving challenging behavior, the evaluation form is being used

□ In 25-50 % of the cases involving challenging behavior, the evaluation form is being used

□ In less than 25 % of the cases involving challenging behavior, the evaluation form is being used

□This form is never used

Question 9

Did the use of the detection tool lead to detection of new cases?

- No
- Yes

If you answered “yes”, could you give examples of cases that have been detected through the use of the detection tool?

Question 10

Were there any barriers for implementation of the care program?

Question 11

How was the support of the management with regards to implementing the care program (in a positive or negative sense)?

Question 12

What were your expectations of the care program before implementation?

Question 13

Would you recommend the use of the care program to co-workers inside or outside your organization?

Question 14

Did you notice any effects of the care program (on the unit and/or on individual residents)?

Questions 15

What are the advantages of working according to the care program?

Question 16

What are the disadvantages of working according to the care program?

Question 17

Could you describe some examples of success you achieved when working with the care program?

Question 18

To what extent are you satisfied with the implementation of the care program?

- Not satisfied
- Hardly satisfied
- Somewhat satisfied
- Satisfied
- Completely satisfied

Question 19

To what extent are you satisfied with the care program itself?

- Not satisfied
- Hardly satisfied
- Somewhat satisfied
- Satisfied
- Completely satisfied

Question 20

Can the care program be used in the available time?

- Yes
- No
- Other:_________________

Question 21

Are there any parts or steps missing in the structure of the care program?

Question 22

Are there any parts or steps redundant in the structure of the care program?

Question 22

Could you name any other points for improvement of the care program?

________________________End of the Questionnaire________________
